# Protein biosynthesis, a target of sorafenib, interferes with the unfolded protein response (UPR) and ferroptosis in hepatocellular carcinoma cells

**DOI:** 10.18632/oncotarget.23843

**Published:** 2018-01-03

**Authors:** Chloé Sauzay, Christophe Louandre, Sandra Bodeau, Frédéric Anglade, Corinne Godin, Zuzana Saidak, Jean-Xavier Fontaine, Cédric Usureau, Nathalie Martin, Roland Molinie, Julie Pascal, François Mesnard, Olivier Pluquet, Antoine Galmiche

**Affiliations:** ^1^ Laboratoire de Biochimie, Centre de Biologie Humaine, CHU Amiens Sud, France; ^2^ EA CHIMERE, Université de Picardie Jules Verne, Amiens, France; ^3^ Laboratoire de Pharmacologie, Centre de Biologie Humaine, CHU Amiens Sud, France; ^4^ Laboratoire d’Oncobiologie Moléculaire, Centre de Biologie Humaine, CHU Amiens Sud, France; ^5^ EA3900, Biologie des Plantes et Innovation, UFR de Pharmacie, Amiens, France; ^6^ Université de Lille, Institut Pasteur de Lille, CNRS UMR8161, M3T: Mechanisms of tumorigenesis and Targeted Therapies, Lille, France

**Keywords:** hepatocellular carcinoma, sorafenib, translation, unfolded protein response (UPR), ferroptosis

## Abstract

Sorafenib is the first line treatment for advanced hepatocellular carcinoma (HCC). We explored its impact on the proteostasis of cancer cells, *i.e.* the processes that regulate the synthesis, maturation and turn-over of cellular proteins. We observed that sorafenib inhibits the production of the tumour marker alpha-foetoprotein (AFP) in two different HCC cell lines, an effect that correlated with a radical inhibition of protein biosynthesis. This effect was observed at clinically relevant concentrations of sorafenib and was not related to the effect of sorafenib on the transport of amino acids across the plasma membrane or the induction of the unfolded protein response (UPR). Instead, we observed that sorafenib inhibits translation initiation and the mechanistic target of rapamycin (mTOR) signaling cascade, as shown by the analysis of phosphorylation levels of the protein 4EBP1 (eukaryotic translation initiation factor 4E binding protein 1). We explored the consequences of this inhibition in HCC cells. We observed that overall sorafenib is a weak inducer of the UPR that can paradoxically prevent the UPR induced by tunicamycin. We also found no direct synergistic anticancer effect between sorafenib and various strategies that inhibit the UPR. In agreement with the possibility that translation inhibition might be an adaptive stress response in HCC cells, we noted that it protects cancer cell from ferroptosis, a form of oxidative necrosis. Our findings point to the modulation of protein biosynthesis and mTOR signaling as being important, yet complex determinants of the response of HCC cells to sorafenib.

## INTRODUCTION

Hepatocellular carcinoma (HCC), the most frequent form of primary liver tumour, remains a major cause of cancer-associated deaths worldwide [1]. Despite its relatively modest efficacy, sorafenib is currently the standard-of-care for the medical treatment of advanced stages of HCC [1]. Sorafenib is a multikinase inhibitor directed against the RAF kinases and several receptor tyrosine kinases (RTK) present at the surface of cancer cells [2]. The precise molecular mechanisms by which sorafenib exerts its clinical efficacy remain unclear. Understanding the mode of action of sorafenib could lead to the identification of important therapeutic targets in HCC, and could potentially also help in treatment personalization [3, 4].

In addition to its direct inhibitory effect on the kinome of cancer cells, recent studies suggest that sorafenib is a drug that potently alters cancer cell proteostasis, *i.e.* the processes that regulate the synthesis, maturation and turn-over of cellular proteins [5]. Sorafenib hinders macro-autophagy and reduces the levels of ubiquitylated proteins in the cell, *i.e.* two mechanisms that account for the regulated turn-over of proteins in eukaryotic cells [6–8]. Recently, sorafenib was shown to inhibit the transport of selected amino acids across the cell plasma membrane through its interaction with the Xc(-) transporter [9]. Sorafenib can also inhibit the folding of nascent proteins, through its ability to interact with the heat shock proteins HSP70 and HSP90, two essential protein chaperones that are implicated in the folding of an array of proteins produced by eukaryotic cells [10]. More recently, Adjibade *et al.* reported that sorafenib also promotes the formation of stress granules*, i.e.* cytoplasmic bodies formed under conditions of stalled translation in cancer cells [11]. Sorafenib therefore appears to be potentially able to interfere with all steps of protein production, chaperoning, folding and turn-over in cancer cells. Protein biosynthesis is a central metabolic pathway in eukaryotic cell physiology [12]. Tumour cells depend on active translation for their sustained replication and biomass production [13]. The translation machinery is a potential therapeutic target and a promising source of biomarkers for the follow-up of tumour responses to medical treatments [13–15]. While sorafenib has been reported to inhibit protein synthesis and lead to the formation of stress granules in HCC cells [11], a link to translation regulation has not yet been established. It is also unclear to which extent the inhibition of protein synthesis relates to the anti-oncogenic efficacy of sorafenib, and in particular to its effect on the two essential kinases ERK and mTOR (mechanistic target of rapamycin) [16].

A large fraction of the proteome of eukaryotic cells transits through the secretory compartment. At this cellular level, eukaryotic cells apply a regulatory mechanism known as the unfolded protein response (UPR) [17, 18]. The UPR is a homeostatic response activated when the folding and the maturation of secreted proteins are compromised, especially in the endoplasmic reticulum (ER) [17]. Three branches of the UPR have been identified in eukaryotic cells, each one defined by its main molecular protein regulator: PKR-like ER kinase (PERK), inositol-requiring enzyme-1α (IRE-1α) and activating transcription factor-6 (ATF6) [17]. Sorafenib applied as a single agent was found by us and others to activate the PERK and IRE-1α branches of the UPR in HCC cells [19, 20]. The kinase PERK is able to phosphorylate the eukaryotic translation initiation factor 2α (eIF2α) and potentialy interferes with the initiation phase of protein translation. The protein IRE-1α is a nuclease whose main reported substrate is the transcription factor X-box protein-1 (XBP1) mRNA. The cleavage of XBP1 mRNA generates a transcriptionaly-active splice variant of XBP1 (sXBP1) [17]. While the UPR is gaining increasing recognition as a contributor to carcinogenesis and a determinant of cancer cell response to various cancer therapeutics [21, 22], it remains unclear how it influences the response of HCC cells to sorafenib.

Proteostasis and redox homeostasis are interconnected branches of cellular metabolism. Notably, the availability of the amino acid cysteine is a limiting factor for the synthesis of gluthathione (GSH), one of the main intracellular redox buffers [23]. We and others have found that sorafenib induces ferroptosis, a new form of regulated non-apoptotic cell death, in various cancer cells [9, 24–26]. The defining feature of ferroptosis is the induction of massive peroxidation of membrane lipids leading to the rupture of plasma membrane continuity [27, 28]. The recognition that ferroptosis is a specific form of regulated necrosis that is potentially applicable to the elimination of cancer cells has raised some interest in its regulation [27–29]. Sorafenib is currently one of the few clinically-approved drugs reported to be able to induce ferroptosis [9, 24, 25]. A better understanding of the regulation of ferroptosis induced by sorafenib offers interesting perspectives in terms of identification of predictive biomarkers, and could ultimately help in the repurposing of this drug.

In the present study, we explored the regulation of proteostasis of HCC cells exposed to sorafenib. In a previous study, we have reported the use of the tumour marker alpha-foetoprotein (AFP), as a reporter of tumour cell proteostasis [20, 30]. We therefore measured the production of AFP and performed various assays to monitor in parallel the anti-oncogenic efficacy of sorafenib, its impact on cellular amino acids and redox metabolism, the regulation of protein biosynthesis and the UPR.

## RESULTS

### Regulation of the production of AFP in HCC at the protein synthesis level

**Figure 1:**
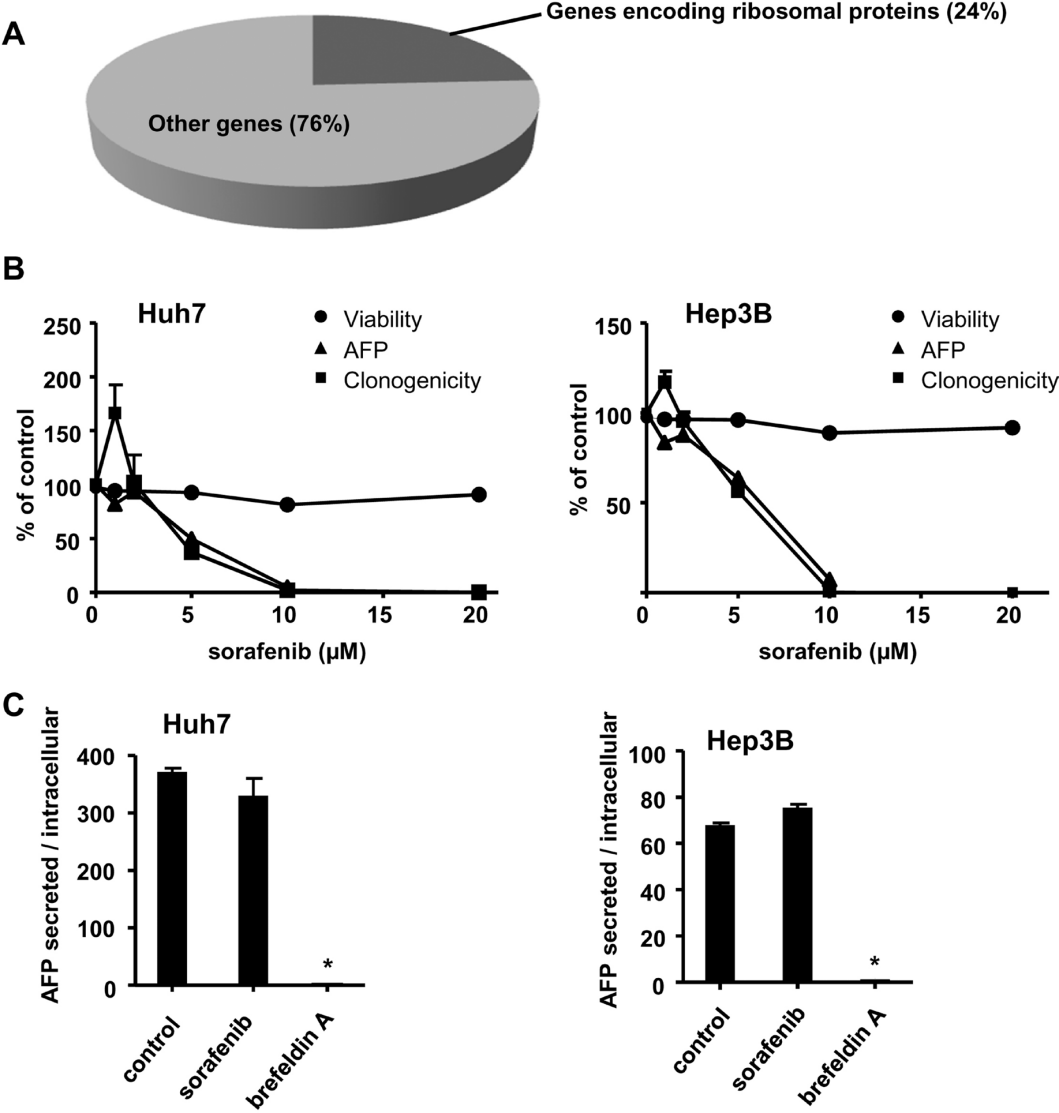
Sorafenib alters tumour cell proteostasis, cell viability and the clonogenic growth of HCC cells **(A)** we present an analysis of the genes whose mRNA expression levels correlate with AFP mRNA in HCC tumours. Data were extracted from the HCC cohort of the TCGA consortium (*n* = 360 patients). Out of the 187 genes that correlated with AFP (Pearson R^2^ > 0.3, *p* < 0.05), 44 (24%) encode ribosomal proteins. (**B**) The human HCC cell lines Hep3B and Huh7 were exposed to increasing concentrations of sorafenib (1-20 µM). We assessed the clonogenic growth of HCC cells exposed to the indicated concentrations of sorafenib for 15 days. In parallel, we measured the concentration of AFP produced in the cell culture medium and cell viability (measured using the Trypan blue exclusion assay) after 18h of treatment with sorafenib. The corresponding values were normalized in each case to the control without sorafenib. (**C**) Concentrations of secreted and intracellular AFP were determined and a ratio was calculated after normalization with respect to control conditions. Note that AFP concentrations were determined after 18h of continuous exposure of cells to the indicated drugs. Brefeldin A was applied at a concentration of 5 µg/mL previously found to be non toxic (data not shown). ^*^indicates *p* < 0.05 compared to control, using Student’s *t* test.

Our previous study showed that AFP is a biomarker that is suitable for probing tumour cell proteostasis and the functionality of the secretion apparatus of HCC cells exposed to sorafenib [20]. To investigate which genes correlate with AFP mRNA expression in HCC, we used data from The Cancer Genome Atlas (TCGA) consortium [31, 32]. We retrieved gene expression data from 360 surgical HCC specimen and identified the genes whose expression levels correlated with AFP *mRNA* (taking Pearson R^2^ > 0.30 as cut-off, and *p* < 0.05). Interestingly, we found that AFP mRNA correlates with the expression levels of several genes that encode ribosomal proteins (Figure 1A). Out of the 187 genes correlated with AFP mRNA, 44 (*i.e.* 24%) encoded ribosomal proteins of the small or large subunits (Figure 1A, data not shown). This observation was specific. The same strategy applied to the *ALB* gene, which encodes albumin, *i.e.* the major secretory protein produced by hepatocytes, identified 252 correlated genes. None of these genes encoded ribosomal subunits (data not shown). This striking coexpression pattern found between the genes encoding AFP and ribosomal proteins prompted us to use AFP to explore tumour proteostasis in HCC cells in the therapeutic context.

We exposed the Huh7 and Hep3B human HCC cell lines, that differ in their response to sorafenib [33], to increasing concentrations of this drug. The HCC cell lines Huh7 and Hep3B produce AFP. In parallel, we measured: i) the levels of AFP produced in the culture supernatant after 18h of exposure to sorafenib; ii) the loss of cell viability after 18h using the trypan blue exclusion assay; iii) the anti-oncogenic activity of sorafenib, measured with a clonogenic assay under conditions of continuous exposure to sorafenib for 15 days (Figure 1B). We found a striking parallel between the relative decrease in the production of AFP and the inhibition of clonogenic growth induced by sorafenib applied at concentrations above 2 µM. This near identical pattern in the effects of sorafenib on AFP production and clone formation was in sharp contrast to the moderate effect of sorafenib on the viability of HCC cells: we saw a loss of cell viability after 18h of sorafenib treatment that was consistently <10% as measured using the Trypan blue exclusion assay, in contrast to the near complete inhibitory effect of sorafenib on AFP production and clonogenic growth (Figure 1B). This effect was partially reversible upon elimination of sorafenib: in Huh7 and Hep3B cells, rinsing off sorafenib resulted in a progressive restoration of AFP secretion that coincided with the restoration of clonogenic growth. The restoration was almost complete after 48h in Hep3B cells, and reached 50% in Huh7 cells (Supplementary Figure 1).

In order to examine the relative contribution of defective production *vs* secretion of AFP, we measured the concentration of this protein in cell supernatants and cell lysates and calculated the ratio (Figure 1C). When cells were exposed to sorafenib, the calculated ratio was found to be not significantly different from control conditions, in sharp contrast to the effect of the secretion blocker brefeldin A, applied at a concentration of 5 µg/mL, which was previously found not to induce significant direct cytotoxicity (data not shown) (Figure 1C). We concluded that sorafenib reduced the production rather than the secretion of AFP. This, combined with the result of our previous study showing little variation in *AFP* mRNA upon exposure of HCC cells to sorafenib [20], led us to conclude that an essential regulation of AFP production occured at the level of the regulation of protein synthesis.

### Sorafenib strongly reduces the levels of protein biosynthesis in HCC cells

**Figure 2:**
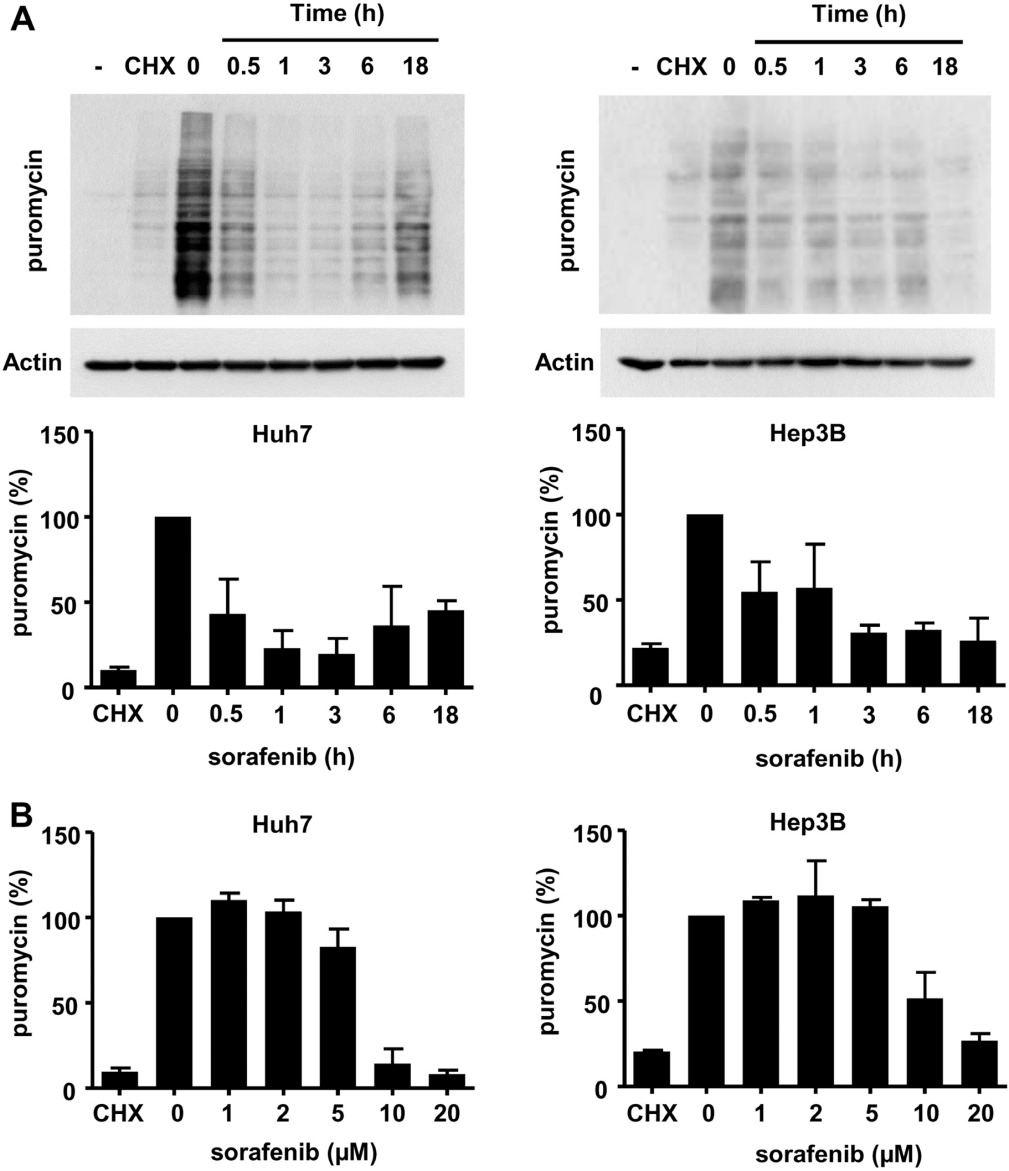
Sorafenib inhibits protein biosynthesis in HCC cell lines (**A**) Time-course analysis. Huh7 and Hep3B cells were exposed to puromycin (25 µg/mL) for 10 minutes and cellular extracts were prepared and analyzed by immunoblotting with an antibody raised against puromycinylated proteins. The condition « - » indicates a control without puromycin. Sorafenib was applied at a concentration of 10 µM for the indicated time. Where indicated, cells were pre-incubated with cycloheximide (CHX, 100 µM for 30 min), an antibiotic that blocks protein translation in eukaryotes. For both cell lines, we present a single representative experiment. The quantification is based on three independent experiments, with the control condition taken as the reference value (100 %). (**B**) Increasing concentrations of sorafenib were applied for one hour to Huh7 and Hep3B cells, and the amount of puromycinylated protein was determined as previously indicated. The quantification is based on three independent experiments, with control conditions taken as reference (100 %).

We measured the incorporation of low concentrations of puromycin into nascent proteins in order to determine the levels of global protein synthesis [34] in Huh7, Hep3B, HepG2 and PLC/PRF5 cells (Figure 2, Supplementary Figure 2). We found that sorafenib exerted a strong inhibitory effect on protein biosynthesis in all HCC cells, which was comparable in its extent to the effect of cycloheximide (100 µM), an antibiotic that inhibits translation and that was used here as a reference (Figure 2, Supplementary Figure 2). This effect of sorafenib was rapid, since protein biosynthesis was reduced by more than 50% after 30 min of exposure to sorafenib in all HCC cells (Figure 2A, Supplementary Figure 2). Huh7 and Hep3B cells however differed in their kinetics, since protein biosynthesis levels partially recovered after 18h in Huh7 cells (Figure 2A). This inhibitory effect was observed at a concentration of 10 µM (Figure 2B), *i.e.* close to the concentrations measured in the serum of HCC patients receiving sorafenib [35]. We found a similar inhibitory effect of sorafenib on protein biosynthesis in immortalized human hepatocytes (IHH), normal human dermal fibroblasts (NHDF) and normal human epidermal keratinocytes (NHEK) (Supplementary Figure 3).

**Figure 3:**
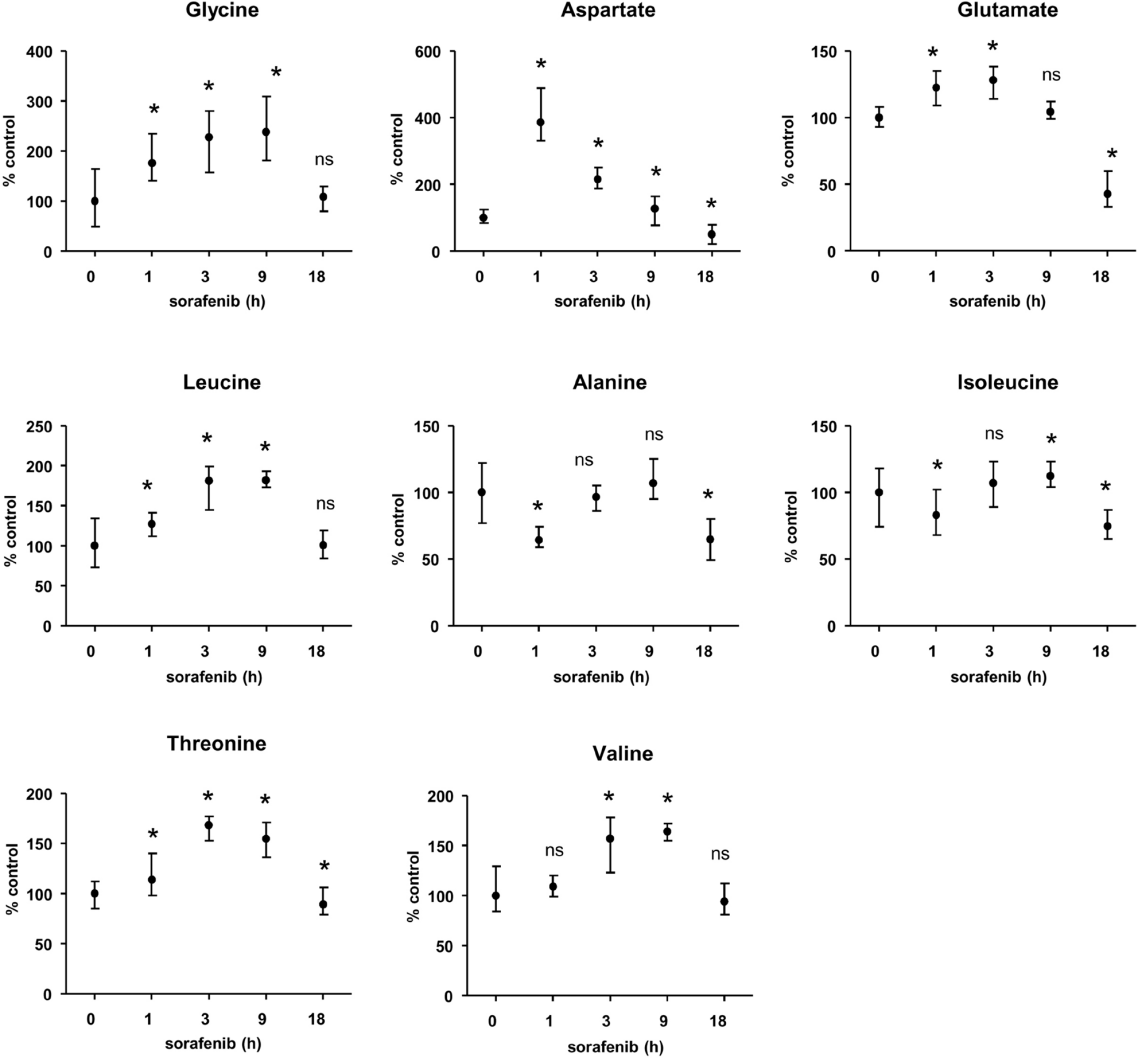
Intracellular concentrations of amino acids in HCC cells exposed to sorafenib Huh7 cells were exposed to sorafenib at various time points (1, 3, 9, 18 h) and the intracellular concentrations of amino acids were measured based on the nuclear magnetic resonance (NMR) spectrum analysis. The results are presented as average of three independent experiments, after normalization with respect to control conditions. ^*^indicates *p* < 0.05 compared to control and n.s. indicates the lack of significant difference compared to control using Student’s *t* test.

We reasoned that sorafenib might inhibit protein biosynthesis via its ability to alter the transport of amino acids across the plasma membrane of HCC cells [9]. We therefore examined the effect of sorafenib on the intracellular content of amino acids using NMR (Figure 3). Huh7 cells were exposed to 10 µM sorafenib for 1, 3, 9, and 18h followed by preparation of cellular extracts. Except for alanine, a significant increase in a number of different amino acids was seen over time (e.g. Gly, Asp, Leu, Thr). This observation strongly suggested that the availability of amino acids was not limiting in this context, and was consistent with the possibility that it resulted from the inhibition of protein biosynthesis induced by sorafenib (Figure 3). In order to nevertheless explore the possibility that sorafenib might block protein biosynthesis by selectively inhibiting the plasma membrane amino acid transporter Xc(-) and the availability of the amino acid cysteine, we tested the effect of the compound N-acetyl cysteine (NAC), a chemical precursor of cysteine [26]. We found that the addition of NAC at a concentration of 10 mM, previously reported by us to radically prevent oxidative stress induced by sorafenib [26], did not revert the inhibition of protein biosynthesis (Supplementary Figure 4). The inhibition of protein biosynthesis therefore appeared to be a specific effect of sorafenib that was apparently neither causally related to oxidative stress nor to the alteration of the cellular amino acid metabolism.

### Sorafenib inhibits mTOR signaling and decreases the initiation of translation

**Figure 4:**
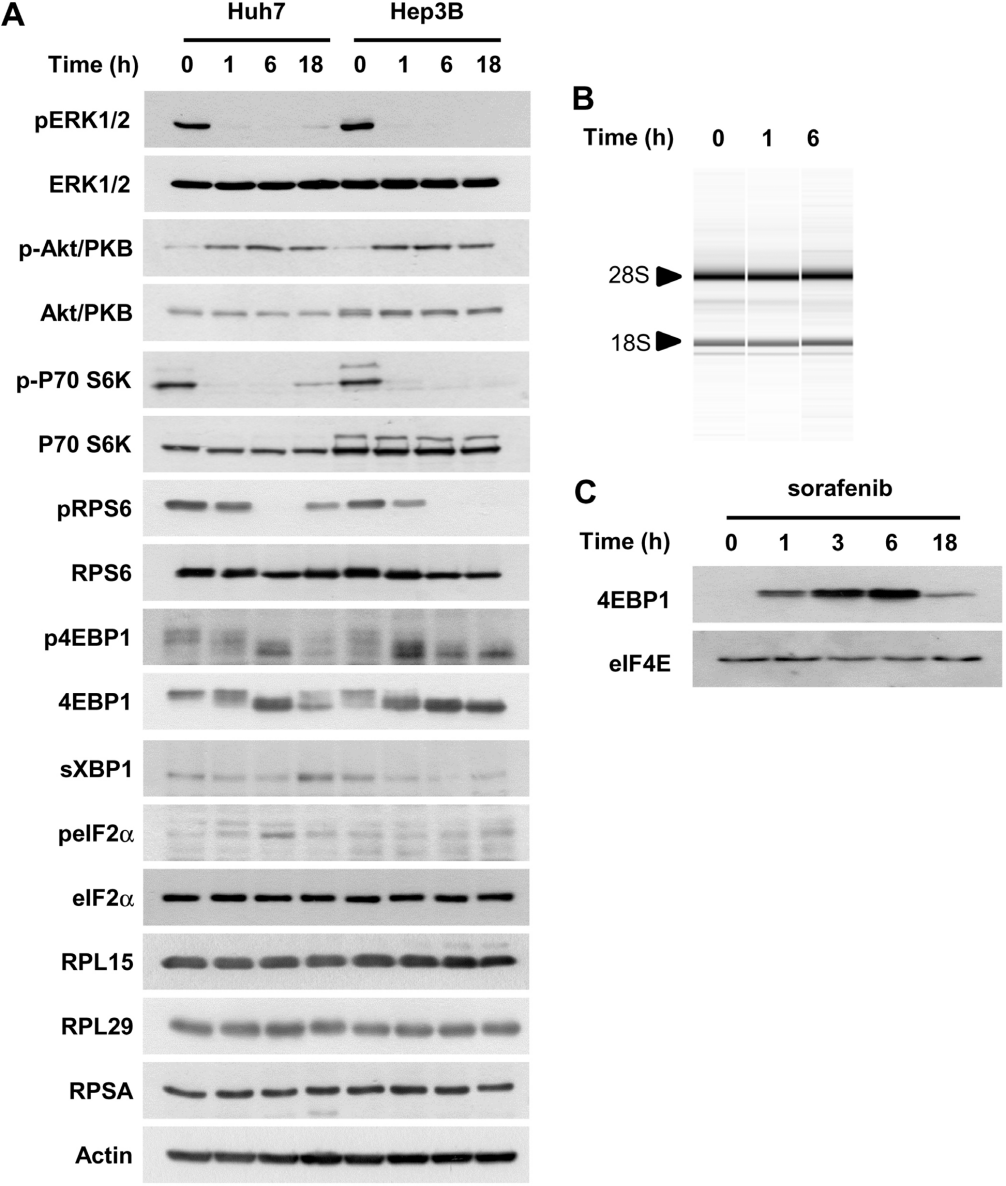
Sorafenib inhibits mTOR signaling and decreases translation initiation in HCC cells (**A**) cellular extracts obtained from Huh7 and Hep3B cells exposed to 10 µM sorafenib were analysed by immunoblotting for their content of the indicated markers. Note that ERK1/2 appears as a single band, due to the predominant detection of ERK2 in the corresponding cells. (**B**) RNA was prepared from Huh7 cells exposed to sorafenib (10 µM, 1 h and 6 h). The two ribosomal RNAs, 28S and 18S, are indicated with arrowheads. (**C**) a cap pull-down assay was performed with m7-GTP sepharose beads, using cellular extracts prepared from Huh7 cells exposed to 10 µM sorafenib for the indicated time.

In order to address the possible mechanism(s) for the inhibition of protein biosynthesis, we carried out an immunoblot analysis using cell extracts prepared from all four HCC cell lines exposed to sorafenib (Figure 4A, Supplementary Figure 5 and 6). We explored the expression levels of the components of the oncogenic kinase ERK and mTOR. We also explored the activation status of the PERK and IRE-1α sensors of the UPR and a number of protein components of the ribosomes (ribosomal protein large subunits RPL5, RPL11, RPL15 and RPL29, and ribosomal protein small subunits RPSA and RPS6), as well as some of the main protein chaperones of the ER and components of the translation initiation complex (Supplementary Figure 6). In Huh7 cells, the levels of the 18S and 28S ribosomal RNA (rRNA) were found to remain stable (Figure 4B). We observed that sorafenib inhibits mTOR signaling, as shown by the analysis of the phosphorylation levels of the proteins 4EBP1 (eukaryotic translation initiation factor 4E binding protein 1), S6K (p70 S6 kinase) and RPS6 (40S ribosomal protein S6) in Huh7, Hep3B (Figure 4A) and HepG2 cells (Supplementary Figure 5). Sorafenib had a more complex effect on PLC/PRF5 cells, since we observed an increase in S6K phosphorylation and simultaneously a reduction in 4EBP1 phosphorylation (Supplementary Figure 5). In all four HCC cell lines, we found that sorafenib increases the phosphorylation levels of Akt/PKB (Figure 4A, Supplementary Figure 5). These findings suggest that the effect of sorafenib on protein biosynthesis correlates with the phosphorylation of 4EBP1, downstream of mTOR signaling.

The effect of sorafenib on 4EBP1 was confirmed by a cap-pull down assay using m7-GTP beads to isolate cap-binding proteins such as 4EBP1 or eIF4E. This assay showed an increased association of m7-GTP with the inhibitory factor 4EBP1 (Figure 4C). The kinetics of this association mirrored the effect of sorafenib on protein biosynthesis, suggesting that sorafenib is able to inhibit translation at the initiation stage. In order to examine the contribution of the two kinase cascades (RAF-MEK-ERK and mTOR) whose activation is hampered by sorafenib, we examined the effect of trametinib and rapamycin on global protein synthesis levels in Huh7 cells. Rapamycin selectively blocked mTOR signaling in HCC cells, while trametinib selectively inhibited the ERK cascade in these cells (Supplementary Figure 7). Rapamycin inhibited global protein synthesis in HCC cells, while the inhibition of ERK induced by trametinib did not block protein biosynthesis (Supplementary Figure 7). These findings suggest that the inhibition of signaling downstream of the mTOR kinase at least partially accounts for the effect of sorafenib on protein biosynthesis in HCC cells.

### Protein biosynthesis regulates cellular response to ER stress and the UPR

**Figure 5:**
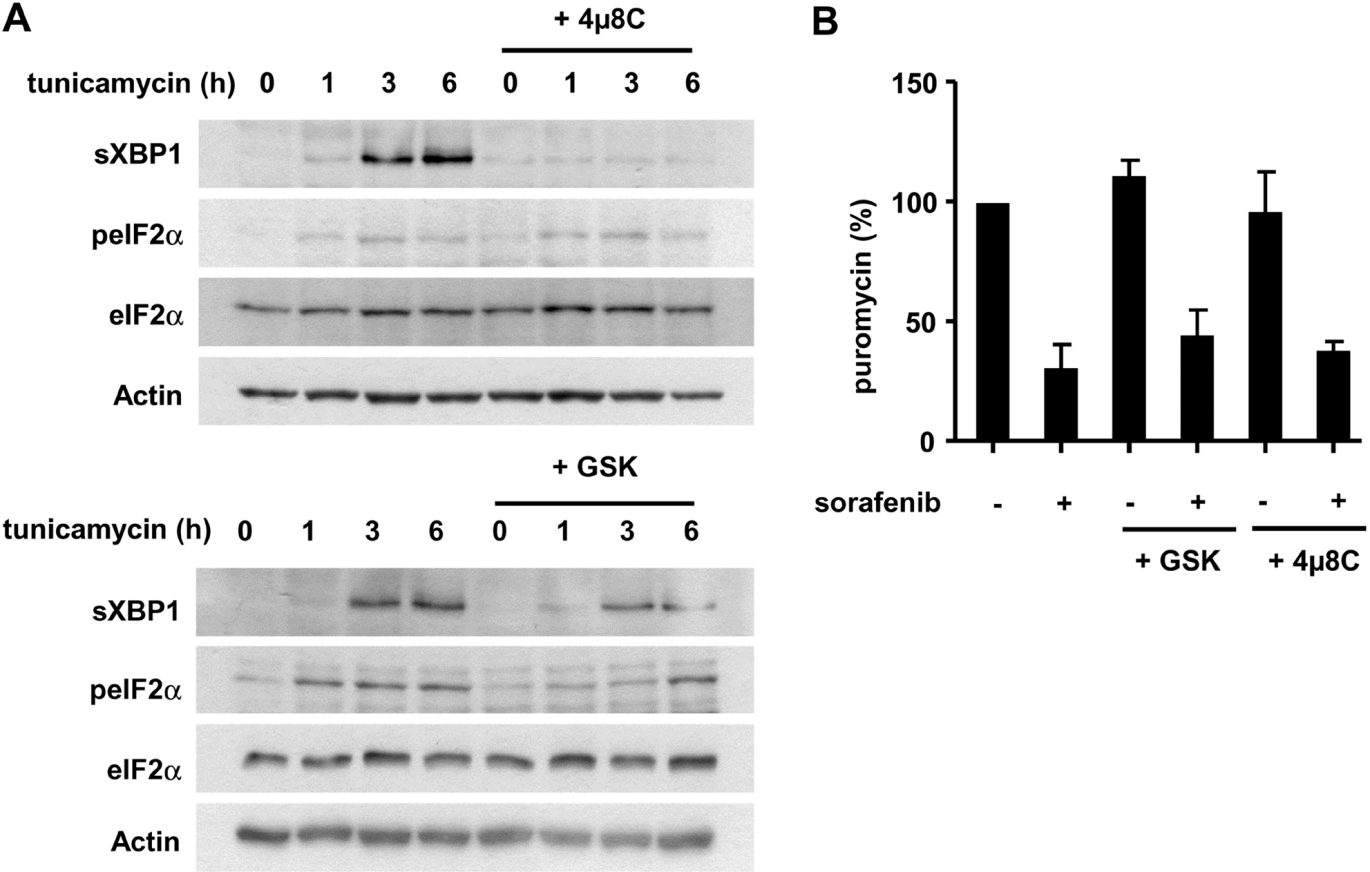
The IRE1α and PERK arms of the UPR are not directly implicated in the inhibition of protein biosynthesis induced by sorafenib (**A**) Huh7 cells were exposed to 4µ8C (10 µM) and GSK2606414 (1µM), two inhibitors directed against the IRE-1α and PERK branches of the UPR, respectively. Tunicamycin, an inducer of ER stress, was applied at a concentration of 10 µM for the indicated period of time. Cellular extracts were prepared and analyzed by immunoblotting with antibodies directed against sXBP1 and the total and phosphorylated form of eIF2α (peIF2α). (**B**) Huh7 cells were preincubated with 4µ8C (10 µM) and GSK2606414 (1µM) for 1 h, and exposed to sorafenib for 1h. An analysis of protein biosynthesis levels was performed with puromycin incorporation and subsequent detection of puromycinylated proteins by immunoblotting. The graph was built from three independent experiments, taking control conditions as reference (100%).

**Figure 6:**
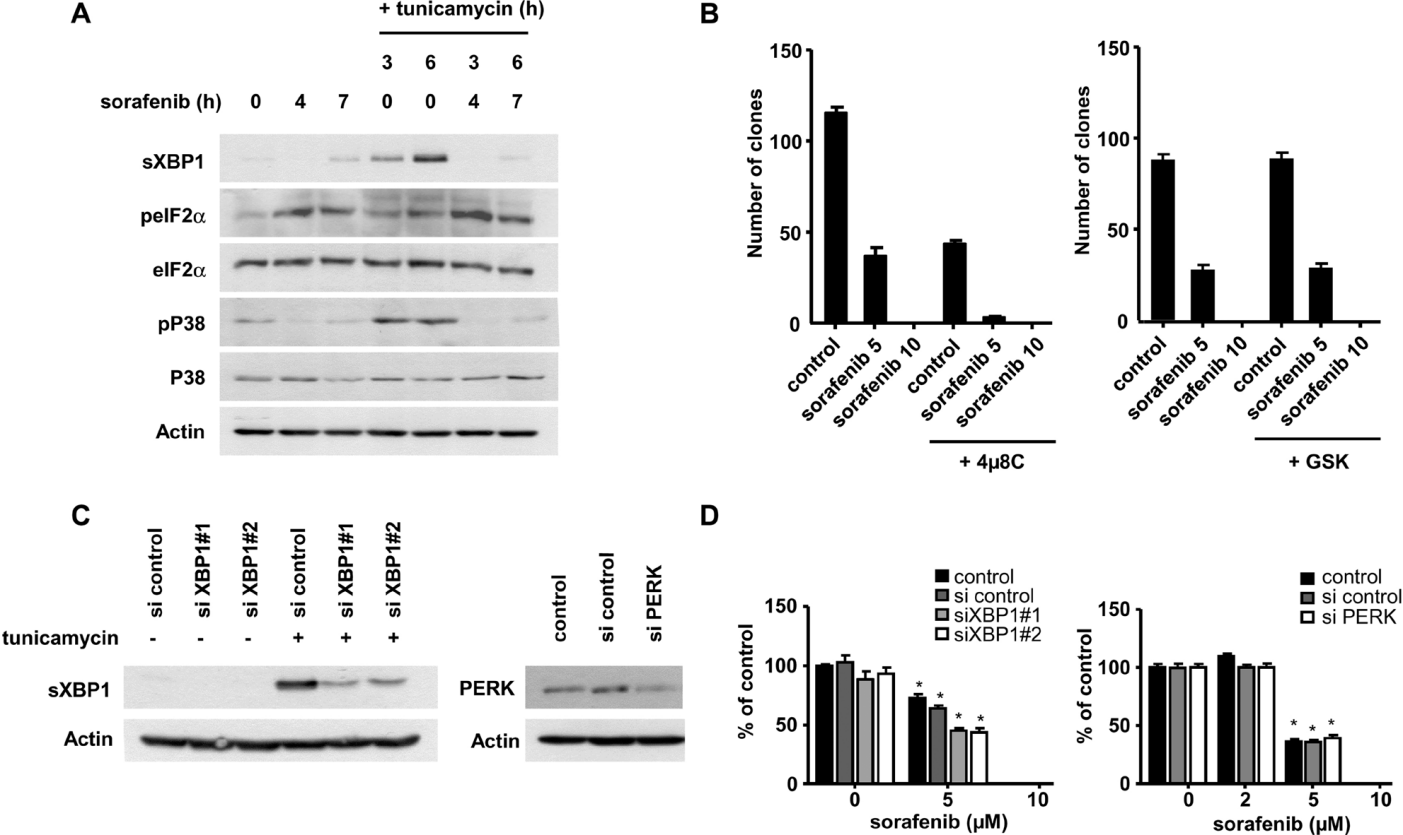
Sorafenib modulates the UPR in HCC cells Lack of pharmacological synergy between sorafenib and antagonists of the UPR *in vitro*. (**A**) Huh7 cells were exposed to sorafenib (10 µM) and tunicamycin (10 μM) for the indicated period of time. Cellular extracts were prepared and analysed for the indicated markers of the UPR. (**B**) A clonogenic assay was carried out using Huh7 cells exposed to sorafenib (5 or 10 µM) with the UPR inhibitors 4µ8C (10 µM) and GSK2606414 (1 µM). The results are from a single representative experiment performed in triplicate. (**C**) Huh7 cells were transfected with two siRNAs targeting distinct regions of XBP-1 and PERK, and exposed to tunicamycin for 6 h as indicated. The efficacy of the knock-down was verified by immunoblot analysis using relevant antibodies. (**D**) A clonogenic assay was performed with Huh7 cells transfected with the indicated siRNA and exposed to sorafenib. The results are from a single representative experiment performed in triplicate.

We decided to further examine how protein biosynthesis and the UPR could functionally interact. We aimed to inhibit the two branches of the UPR previously reported by us and others to be active in HCC cells exposed to sorafenib [19, 20]. The chemical inhibitors 4µ8C and GSK2606414, directed against the nuclease activity of IRE-1α and the kinase activity of PERK [36, 37], were applied on Huh7 cells at pharmacologically-active concentrations (Figure 5A). Neither of the two inhibitors prevented the inhibitory effect of sorafenib on protein synthesis (Figure 5B), suggesting that the UPR was not involved in the inhibition of protein biosynthesis in HCC cells. Conversely, we reasoned that the inhibition of translation could potentially reduce ER stress and the induction of the UPR. We applied sorafenib as a single agent or in the presence of tunicamycin, an inhibitor of N-glycosylation and a potent inducer of ER stress, to Huh7 cells (Figure 6A). This experiment revealed that sorafenib was not only a weak inducer of the UPR compared to tunicamycin, but that it also largely prevented the activation of sXBP1 induced by tunicamycin (Figure 6A). These findings prompted us to directly examine the biological importance of the UPR in the response of HCC cells to sorafenib. We carried out a clonogenic growth analysis using HCC exposed to sorafenib with 4µ8C and GSK2606414 (Figure 6B). The chemical compound GSK2606414 had no significant inhibitory effect on the clonogenic growth of Huh7 cells exposed to sorafenib, but 4µ8C slightly reduced the clonogenic growth of Huh7 cells (Figure 6B). This last effect was however most likely an off-target effect of this compound. Indeed, in an attempt to relate this effect to the inhibition of XBP1s, we used two small interfering RNAs (siRNA) directed against this protein (Figure 6C). We found that a strong reduction in XBP1s expression had only a minor effect on the clonogenic growth of HCC cells under our experimental conditions (Figure 6D). RNA interference directed against PERK kinase had no modulatory effect on the anti-clonogenic efficacy of sorafenib (Figure 6C, 6D). We concluded that sorafenib and antagonists of the UPR had little or no synergistic effect *in vitro*.

### Translation initation as a determinant of cellular susceptibility to oxidative stress and ferroptosis

**Figure 7:**
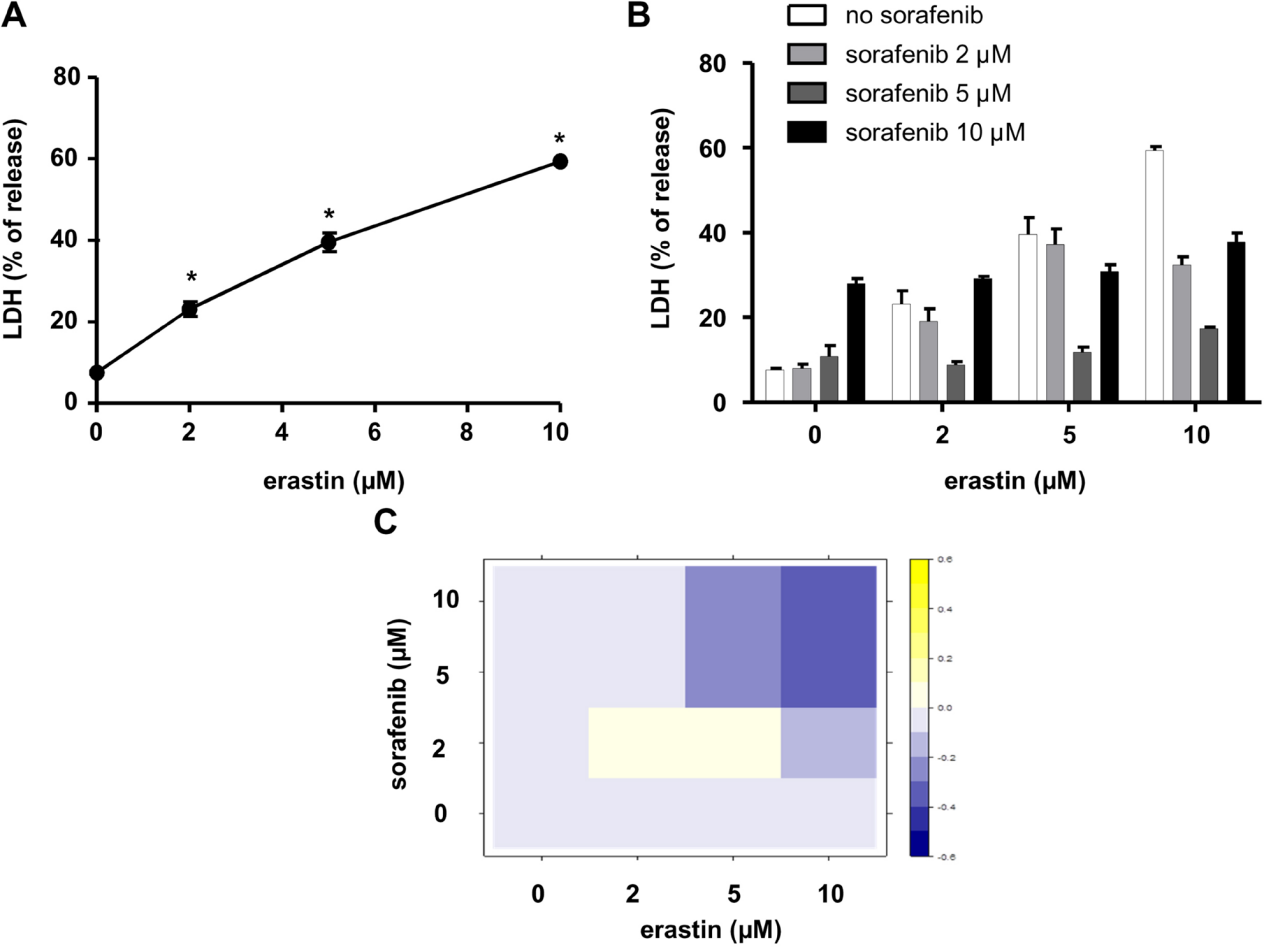
Antagonistic interaction between erastin and sorafenib in their cytotoxicity in HCC cells (**A**) Huh7 cells were exposed to erastin at increasing concentrations for 18 h. The graph shows % released LDH calculated for each condition. ^*^p < 0.05 compared to control conditions using Student’s *t* test. (**B**) Huh7 cells were simultaneously exposed to erastin and sorafenib, applied at the indicated concentrations and maintained for 18 h. The graph shows % released LDH for each condition. (**C)** A Bliss independence analysis was performed using data from panel B. Note that the conditions marked with a blue square indicate an antagonistic interaction between erastin and sorafenib at concentrations > 5 µM in terms of their cytotoxicity.

Next, we addressed the possibility that the regulation of translation initiation by sorafenib may modulate the susceptibility of HCC cells to ferroptosis, a form of regulated necrosis characterized by the occurrence of oxidative stress [29]. We applied erastin, the inducer of reference for this type of cell death to Huh7 cells and measured the % of LDH released in the culture medium under these conditions. We found that erastin induced LDH release in these cells with high efficacy (EC_50_= 5.3 µM) (Figure 7A), in accordance with previous results [24]. We next examined the effects produced by the combined application of sorafenib and erastin over a range of concentrations using Huh7 cells (Figure 7B). Interestingly, at concentrations > 5 µM, sorafenib reduced the levels of cell death induced by erastin by almost two-fold (Figure 7B). A statistical analysis based on the Bliss independence model [38] showed the antagonistic interaction between sorafenib and erastin in their cytotoxicity (Figure 7C).

**Figure 8:**
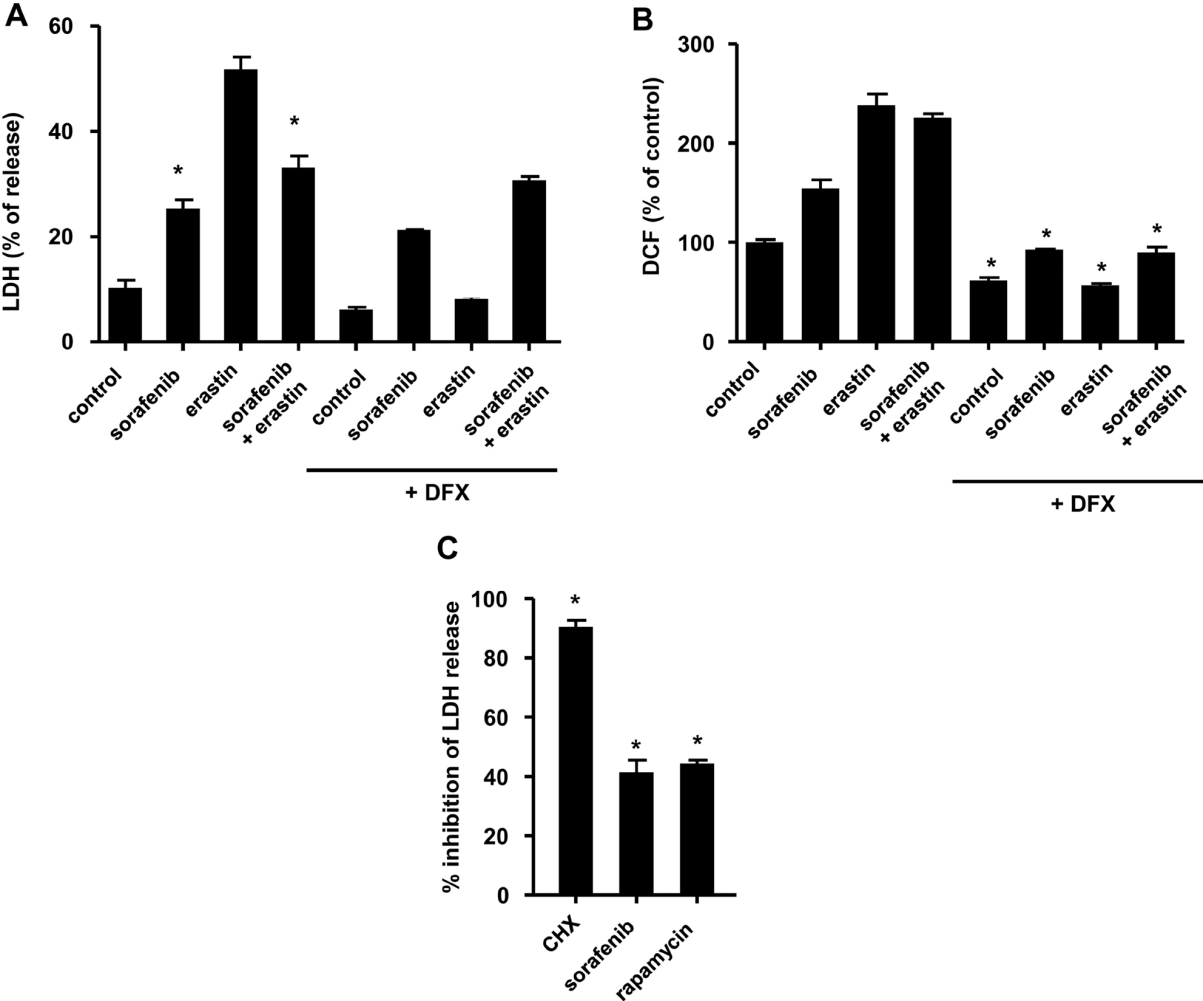
Sorafenib partially protects HCC cells from oxidative stress and ferroptosis induced by erastin (**A**) Huh7 cells were exposed to sorafenib (10 µM) and erastin (5 µM) for 18 h. Deferoxamine (DFX) was simultaneously applied at a concentration of 100 µM, in order to selectively prevent cell death by ferroptosis. The graph shows % released LDH calculated for each condition. ^*^p < 0.05 compared to conditions without sorafenib using Student’s *t* test. (**B**) Fluorescence analysis with CM-DCFDA in Huh7 cells exposed to sorafenib (10 µM) and erastin (5 µM) for 6h. ^*^p < 0.05 compared to equivalent conditions without sorafenib. (**C**) Huh7 cells were exposed to cycloheximide (100 µM), sorafenib (10 µM) and rapamycin (1 µM) applied one hour before erastin (5 µM). The efficacy of each drug as a blocker of ferroptosis is expressed as % inhibition of ferroptosis, based on the LDH release assay measured at 18 h in these conditions. ^*^p < 0.05 compared to the conditions with erastin alone (not shown) using Student’s *t* test.

We confirmed the ferroptotic nature of the cell death observed under our experimental conditions upon cell exposure to erastin by showing that deferoxamine (DFX) had a protective effect when applied simultaneously with erastin at a concentration of 100 µM (Figure 8A). Next, we used the fluorescent redox-sensitive probe CM-DCFDA in order to examine the impact of sorafenib on the redox metabolism of Huh7 cells exposed to erastin. Using this probe, we found that the simultaneous application of erastin and sorafenib (applied at ferroptotic concentrations of 5 µM and 10 µM respectively) did not increase oxidative stress compared to erastin alone (Figure 8B). These results suggested the possibility that translation initiation is a potential determinant of cancer cell sensitivity to ferroptosis. To directly address the possible role of the mTOR kinase in this effect, we compared the efficacy of sorafenib and rapamycin against ferroptosis induced by erastin (5 µM) in Huh7 cells. The efficacy of sorafenib and rapamycin as blockers of ferroptosis was expressed as % of inhibition of ferroptosis, based on the LDH release assay in these conditions (Figure 8C). In this setting, the protective efficacy of sorafenib was found to be comparable to that of rapamycin (Figure 8C).

## DISCUSSION

In the present study, we explored the impact of sorafenib, the medical treatment of reference for HCC, on the proteostasis of cancer cells. We observed that sorafenib strongly inhibits AFP production and protein biosynthesis in different HCC cell lines and a variety of eukaryotic cells (including primary cells). This effect is likely to be a general effect of sorafenib, *i.e.* relatively independent of the genetic background of eukaryotic cells. It was observed at clinically-relevant concentrations of sorafenib and was most likely accounted for by the modulation of translation initiation. Indeed, we found that the effect of sorafenib on protein biosynthesis correlates with the inhibition of 4EBP1 phosphorylation, a well-established target of mTOR cell signaling [39, 40] We explored the consequences of this inhibition of translation, and report here that global protein biosynthesis modulates the UPR in HCC cells. In agreement with the possibility that translation inhibition might be an adaptive stress response in HCC cells exposed to sorafenib, we also observed that it protects cancer cells from ferroptosis, a form of regulated necrosis that is induced by catastrophic perturbation of cellular redox metabolism.

Our study sheds light on the role played by the UPR in HCC cells exposed to sorafenib. The UPR is a homeostatic response that is likely essential for every aspect of malignant transformation, but the details of its regulation are complex and remain poorly known [21]. The IRE-1α and PERK arms of the UPR were recently found to be activated in an animal model of liver carcinogenesis, and the PERK kinase was reported to constitute a possible therapeutic target in this setting [41]. Based on previous reports showing that the IRE-1α and PERK arms of the UPR are activated in HCC cells exposed to sorafenib [19, 20], we examined the possible interactions between sorafenib and the UPR. We found that the activation of the UPR by sorafenib was modest in amplitude compared to the reference compound tunicamycin. Sorafenib was able to reduce the amplitude of the induction of the sXBP1 arm of the UPR induced by an independent ER stress. In this respect, our results are consistent with those of other investigators that have shown that sorafenib blocks the induction of the UPR in HCC cells exposed to proteasome inhibitors [8]. We found little or no benefit of the combination of sorafenib with chemical or genetic strategies targeting the IRE-1α and PERK arms of the UPR, at least *in vitro*. We propose that the inhibition of global protein synthesis, by decreasing the burden of nascent proteins reaching the lumen of the ER, could explain our *in vitro* observations. At this stage however, we cannot rule out the possibility that the UPR could be an interesting target in specific situations encountered *in vivo*, such as hypoxia or perturbations of the nutritional supply.

The inhibition of protein biosynthesis that we have observed in HCC could contribute to the anti-oncogenic efficacy of sorafenib, and at the same time also promote the resistance of HCC cells to cell death induced by various cellular stresses. We observed a striking correlation between AFP production, shown here to reflect the levels of global protein synthesis, and clonogenic growth upon the application of sorafenib to HCC cells *in vitro*. Inhibition of translation is however also well known to be a response to cellular stress [42–45]. We explored here the response of HCC cells to perturbations of the redox metabolism. The finding that sorafenib can both induce ferroptosis as a single agent [9, 24] and protect HCC cells from ferroptosis induced by erastin might at first seem odd. The existence of multiple and partially-antagonistic modes of action of sorafenib is a likely explanation for this apparent paradox. Inhibition of the amino acid membrane transporter X(c)-, responsible for the cellular uptake of the amino acid cystine, most likely accounts for the ability of sorafenib to block the synthesis of reduced glutathione (GSH) and promote ferroptosis [9]. We propose that the inhibition of protein biosynthesis induced by sorafenib or rapamycin could protect HCC cells from ferroptosis by increasing the availability of amino acids for GSH synthesis. This proposition is supported by the findings of previous studies, where the authors observed that protein biosynthesis and GSH compete for cysteine in eukaryotic cells [23, 46]. Our findings could explain why sorafenib is in general a weaker inducer of ferroptosis than the reference compound erastin [25]. Based on our observations, we suggest that translation, under the control of mTOR signaling, is an important determinant of the susceptibility of cancer cells to ferroptosis. Future studies will need to examine the effect of ferroptotic drugs, such as sorafenib, on mTOR signaling. To address the complex regulation of HCC cell proteostasis in the therapeutic context, system biology approaches will likely be required. Indeed, adressing the intricated regulation of redox metabolism, tumour proteostasis and mTOR signaling, together with the complex regulatory loops and functional redundancies that characterize mTOR signaling in cancer cells [47, 48] promises to be challenging.

## MATERIALS AND METHODS

### Cell culture and reagents

Details regarding the provenance of all cell lines used here, the cell culture protocols and a list of all reagents used in this study can be found in the Supplementary Materials and Methods section. Trypan blue exclusion assay was used for the determination of cell viability.

### TCGA data extraction

Gene expression data were extracted from the TCGA HCC cohort (TCGA Provisional, *n* = 360 patients), using the CBioportal web site (http://www.cbioportal.org/) [31, 32]. Expression levels of each mRNA were calculated based on RNA sequencing data and the software RNAseq v2 RSEM (RNA-Seq by Expectation Maximization). For each gene analyzed, we retrieved all genes whose expression levels were found to be significantly correlated (Pearson R^2^ > 0.3 and *p* < 0.05).

### Measurement of AFP concentrations

AFP concentrations were measured in cell culture supernatants and cellular lysates using the Vista Dimension 500 analyser (Siemens) and the corresponding kit recommended for routine clinical practice. To determine the ratio of secreted over intracellular AFP, we measured AFP produced in the cell supernatant after 18h of culture of HCC cells. A subsequent step of cell lysis was performed in an equal volume of lysis buffer in order to determine intracellular AFP concentrations.

### Determination of the levels of protein biosynthesis

The SUnSET technique relies on the incorporation of puromycin into nascent proteins and its subsequent detection with a monoclonal antibody directed against puromycin, and was previously reported by Schmidt *et al.* [33]. Briefly, for each experimental condition, 2X10^5^ cells were exposed to 25 µg/mL puromycin (Sigma) in the cell culture medium for 10 min before lysis. Cycloheximide (Sigma) was used as a negative control and applied at a concentration of 100 µM for 30 min before lysis. Details regarding the cell lysis protocol and processing for the immunoblot analysis are described in a later section.

### Nuclear magnetic resonance (NMR) analysis

Details of sample extraction and NMR acquisition can be found in the Supplementary Materials and methods section.

### Clonogenicity assay

Clonogenic growth was measured as previously described [34]. Briefly, 200 viable cells were counted using a Trypan blue exclusion assay and an automated cell counter Countess (Invitrogen). Cells were seeded and exposed to the indicated conditions. After 15 days of culture, the clones were fixed with methanol, rinsed with PBS, and stained with a Giemsa solution. A blind count was subsequently performed, with each experimental point representing the average value of a triplicate.

### Ferroptosis measurement

The levels of Lactate dehydrogenase (LDH) released in the culture medium were determined using the CytoTox 96 non-radioactive cytotoxicity assay kit (Promega). The results are expressed as % of total LDH released. Total LDH, taken as 100%, was obtained by adding Triton X100 to the cell culture medium at a final concentration of 0.1% for 5 min at room temperature.

### Oxidative stress measurement

Chloro-methyl-dichlorofluorescein diacetate (CM-DCFDA) (Molecular Probes) was applied at 1 × 10^−6^ M in the cell culture medium for 15 min, washed twice with PBS, and cells were lysed in H_2_O. Fluorescence was determined using a RF-5301PC spectrofluorometer (Shimadzu) (excitation: 480 nm; emission: 525 nm). Values were normalized to the protein content of the extract [24].

### Western blots

For each experimental condition, complete cell extracts were prepared in RIPA buffer. Protein concentrations were determined with a BCA kit (Thermo Fisher). A total of 50 µg of protein were precipitated with methanol and chloroform [49]. The proteins were denatured in Laemmli sample buffer, loaded on SDS-PAGE, and transferred to nitrocellulose membranes using standard procedures. Membranes were saturated for 1h in 5% milk in TTBS (Tween 0.05%, NaCl 200 mM, Tris-HCl pH8), then rinsed and incubated overnight with a 1:1000 dilution of each primary antibody (references listed in the Supplementary Materials and methods section). Later, secondary antibodies coupled with HRP (Horse radish peroxidase) were incubated for 1h at a 1:5000 dilution. The ECL reaction was used for detection.

### RNA isolation and measurement

Total RNA was isolated using the TRIzol reagent (Eurobio, Les Ulis, France). RNA concentration and quality were measured using the Agilent RNA 6000 Nano kit and Agilent 2100 Bioanalyzer.

### Cap pull-down assay

Affinity chromatography with 7-methyl-GTP (m7-GTP)-Sepharose 4B (Jena Biosciences) was performed as previously reported [50]. Cells were lysed in a buffer containing 50mM Tris-HCl (pH 7.5), 150 mM NaCl, 1mM EDTA, 1mM EGTA, 1% Triton X100, 0.5% NP-40 with protease inhibitors. For each condition, 250 µg of protein lysate were incubated with 20 µL of m7-GTP beads. After a one hour incubation at 4°C, beads were washed three times in lysis buffer, and protein samples were recovered in Laemmli sample buffer (5 min at 95°C). Samples were subsequently analyzed by immunoblotting [35].

### Statistical analyses

Student’s *t*-test was used as appropriate, and a value of *p* < 0.05 was considered as threshold for significance. Statistical analyses were performed with R3.02. The Bliss independence analysis was performed using the package “Synergy finder” (https://bioconductor.org/packages/release/bioc/html/synergyfinder.html).

## SUPPLEMENTARY MATERIALS FIGURES


