# Supplementary material for: Protein biosynthesis, a target of sorafenib, interferes with the unfolded protein response (UPR) and ferroptosis in hepatocellular carcinoma cells
Source: Oncotarget. 2018 Jan 3;9(9):8400–14. doi: 10.18632/oncotarget.23843 (PMC5823558; doi:10.18632/oncotarget.23843)
Supplement: Supplementary file 1 [file oncotarget-09-8400-s001.pdf]

Protein biosynthesis, a target of sorafenib, interferes with the unfolded protein response (UPR) and ferroptosis in hepatocellular carcinoma cells

SUPPLEMENTARY MATERIALS

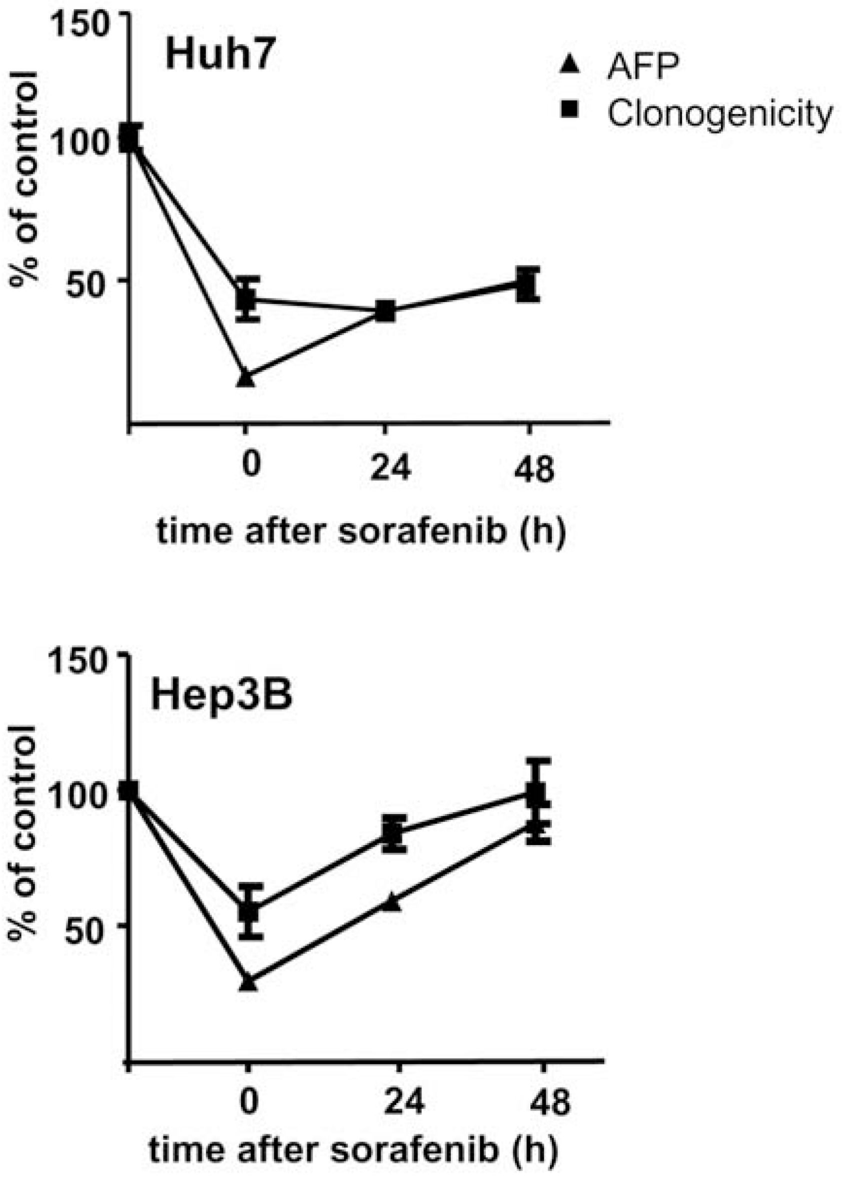

Supplementary Figure 1: Reversibility of the effect of sorafenib on AFP production and clonogenic growth. Huh7 and Hep3B cells were exposed to sorafenib (10 µM) for 18 h. The medium was then replaced with fresh medium without sorafenib. AFP concentrations in the supernatant were measured immediately after stopping the sorafenib treatment, and 24h or 48h later. In parallel, for each condition, a clonogenic assay was carried out in cell culture medium without sorafenib. In each case, the corresponding values were normalized to the control without sorafenib.

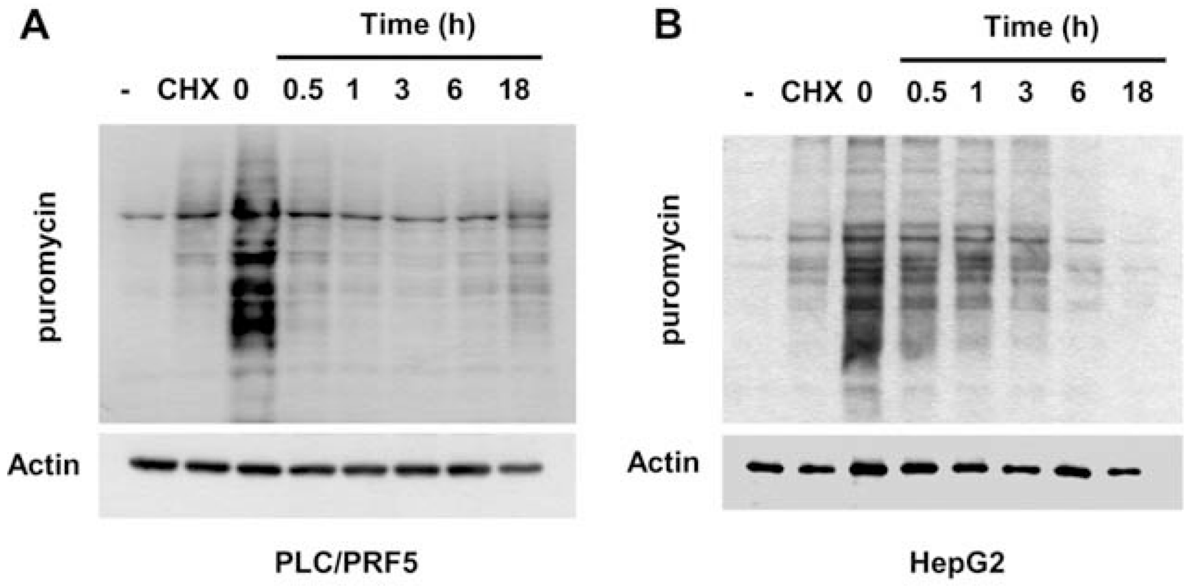

Supplementary Figure 2: Sorafenib inhibits protein biosynthesis in PLC/PRF5 and HepG2 cells. HCC cells were exposed to puromycin (25 µg/mL) for 10 minutes and cell extracts were prepared and analyzed by immunoblotting with an antibody raised against puromycinylated proteins. The condition " – " indicates "control without puromycin". Sorafenib was applied at a concentration of 10 µM for the indicated time. Where indicated, cells were pre-incubated with cycloheximide (CHX, 100 µM for 30 min).

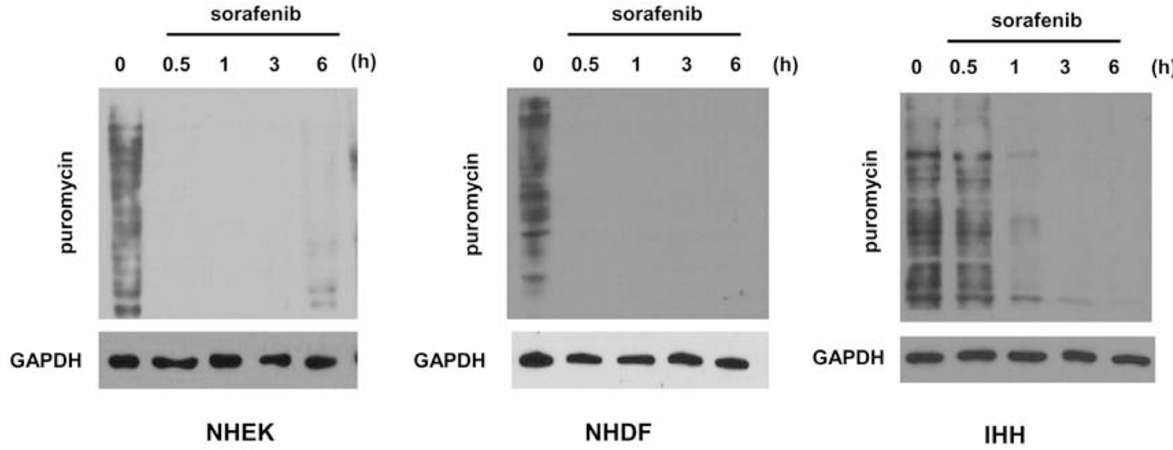

Supplementary Figure 3: Sorafenib inhibits protein biosynthesis in normal human epidermal keratinocytes (NHEK), normal human dermal fibroblasts (NHDF) and immortalized human hepatocytes (IHH). Each cell type was exposed to 10 µM sorafenib applied for the indicated time. Puromycin (25 µg/mL) was applied for 10 minutes before the preparation of cellular extracts, and puromycinylated proteins were later detected by immunoblotting with a specific antibody.

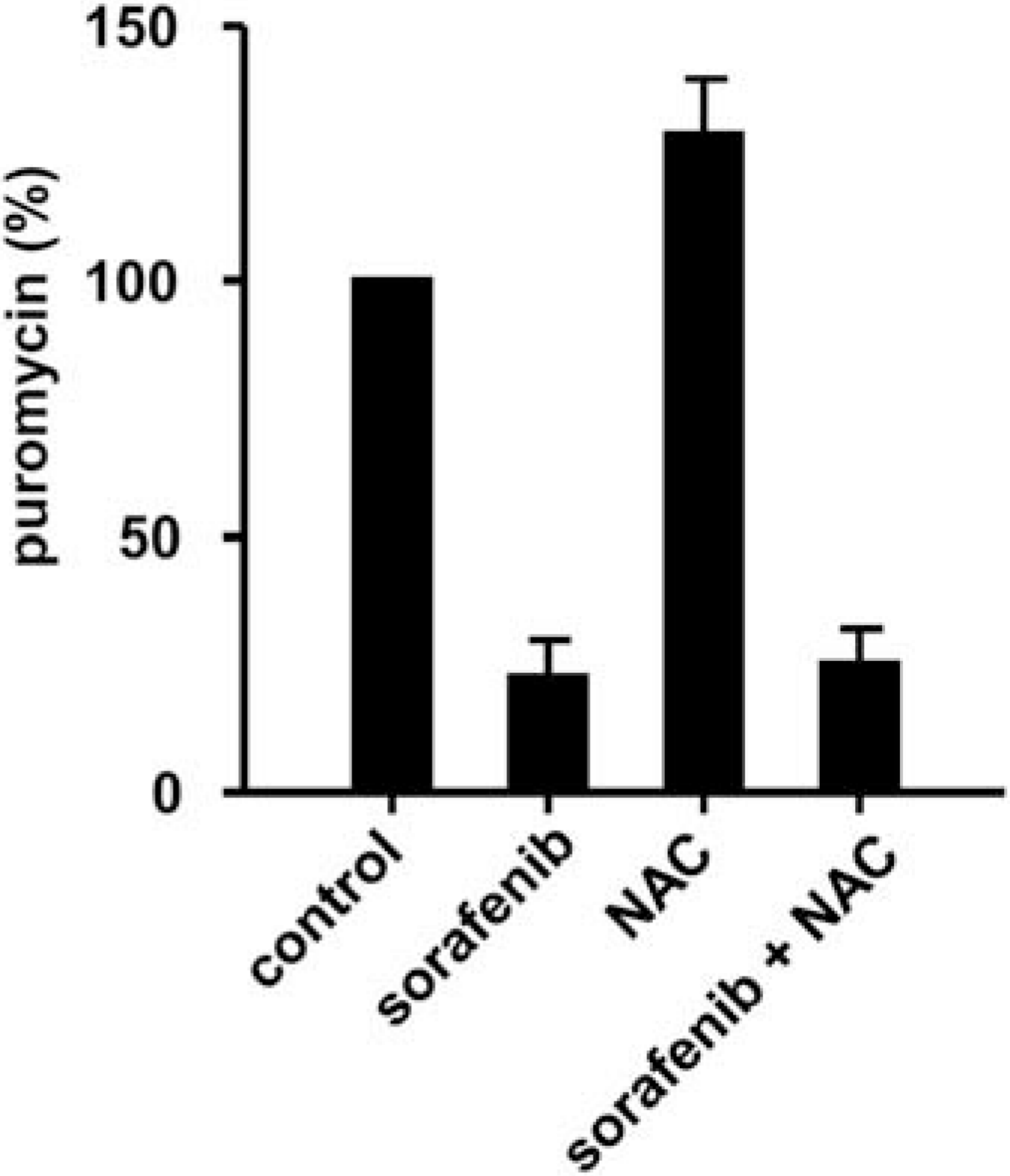

Supplementary Figure 4: N-Acetyl Cystein (NAC) does not prevent the inhibition of protein neosynthesis by sorafenib. Huh7 cells were preincubated for 1 h with NAC (10 mM) before adding sorafenib (10 µM) for 1h. Cells were exposed to puromycin and processed as indicated in the Materials and methods section in order to analyse global levels of protein neosynthesis. The quantification is based on three independent experiments, with control conditions taken as reference (100 %).

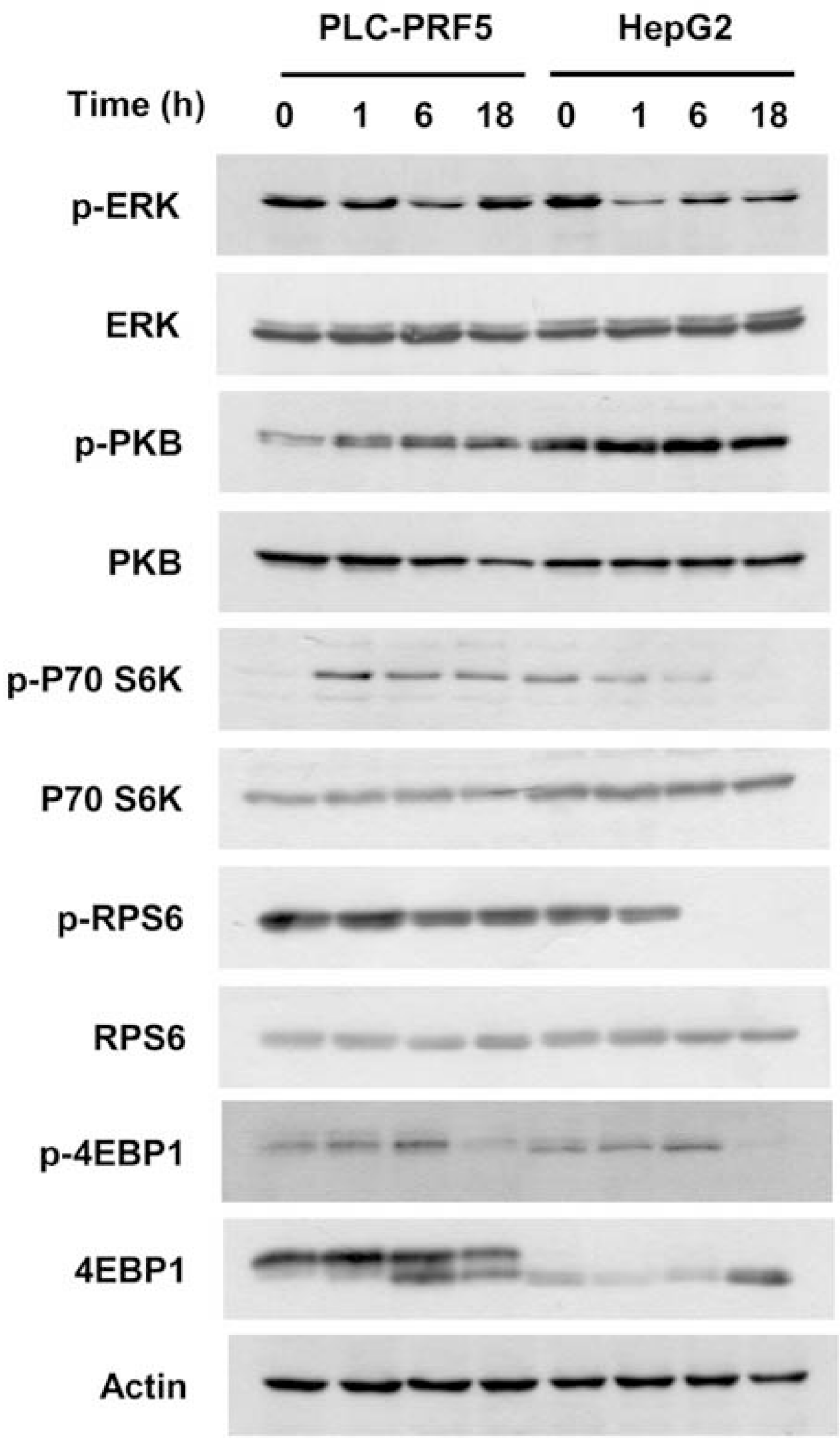

Supplementary Figure 5: mTOR signaling and the regulation of translation initiation in PLC/PRF5 and HepG2 cells. Cell extracts obtained from PLC/PRF5 and HepG2 cells exposed to 10 μM sorafenib were analysed by immunoblotting for their content of the indicated markers.

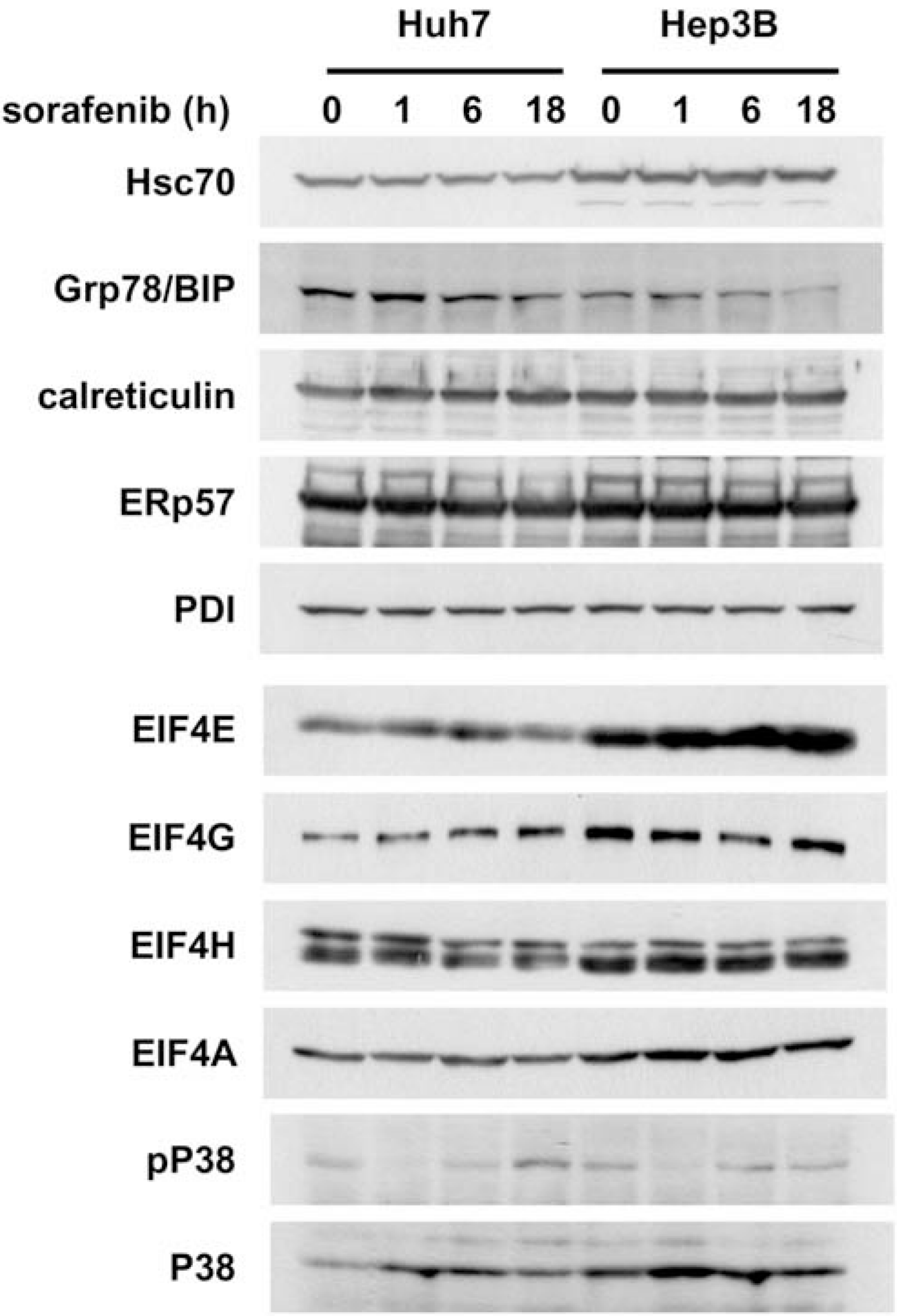

Supplementary Figure 6: Time course analysis of the effect of sorafenib on ER chaperones and translation initiation factors. The expression of the indicated proteins was analysed by immunoblotting. Cell extracts were prepared from Huh7 and Hep3B cells exposed to 10µM sorafenib for the indicated times

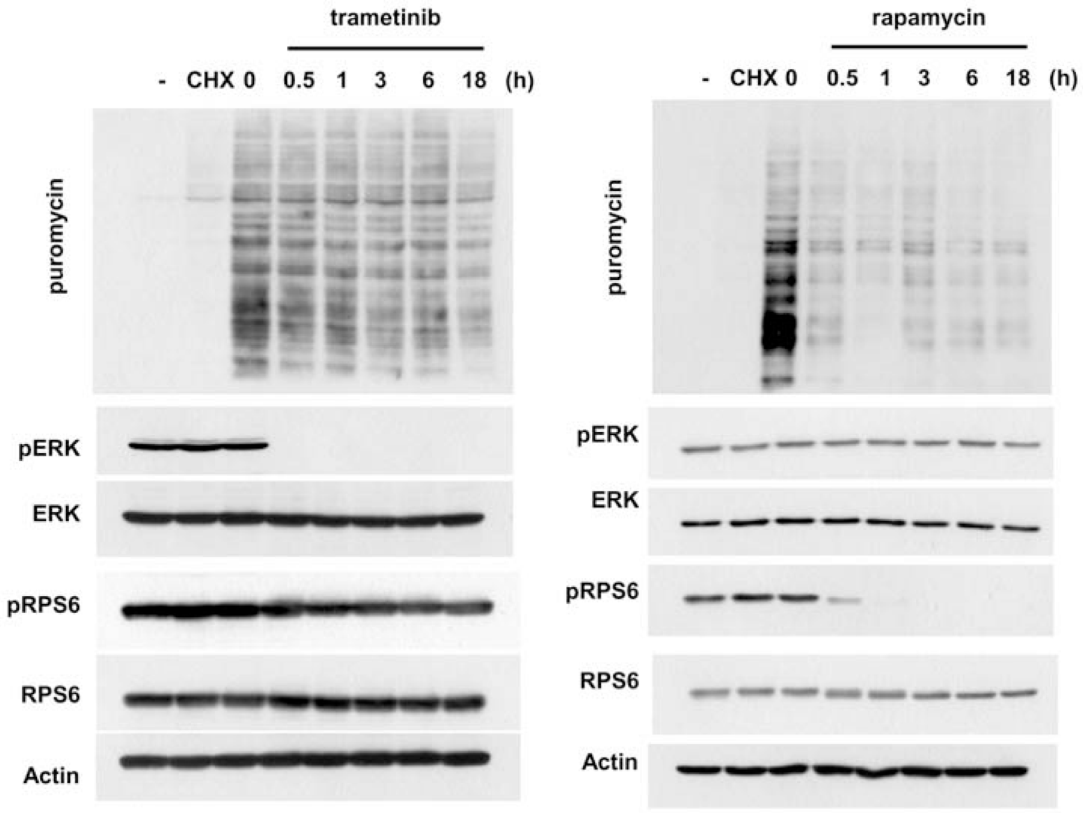

Supplementary Figure 7: Time course analysis of the effect of trametinib or rapamycin on oncogenic kinases and levels of global protein synthesis. Huh7 cells were exposed to trametinib (1 µM), a MEK inhibitor or rapamycin (1 µM), an mTOR inhibitor for the indicated times. Cells were exposed to puromycin and processed as indicated in the Materials and methods section in order to analyse global levels of protein neosynthesis. The condition "-" indicates "control without puromycin". Where indicated, cells were pre-incubated with cycloheximide (CHX, 100 µM for 30 min). The expression levels of oncogenic kinases were also analysed by immunoblotting from the same cell extracts.

Cell culture & cell lines

The HCC cell lines used in this study (Huh7, Hep3B, PLC/PRF5 and HepG2) were obtained from Dr. Wychowski (Institut de Biologie de Lille, France) and were authenticated using profiling of short tandem repeats at 16 loci (LGC Standards, Strasbourg, France). Cells were cultured in Dulbecco Modified Eagle's Medium (DMEM) supplemented with 10% fetal calf serum (Jacques Boy), 2 mM glutamine, 100 µg/mL penicillin and streptomycin.

Human immortalized hepatocyte (IHH) cells were cultured in Williams E medium (Life Technologies), supplemented with 10% fetal bovine serum (Life Technologies), 100 U/mL penicillin (Life Technologies), 100 µg/mL streptomycin (Life Technologies), 20 mU/mL insulin (Novo Nordisk) and 50 nM/L dexamethasone (Sigma). IHH cells were cultured in 0.1% gelatin-coated culture flasks (gelatin from porcine skin, Sigma). Normal human dermal fibroblasts (NHDF) were purchased from Clonetics (Basel, Switzerland). One skin donor was used (referred to as F6MC1, a 6 years-old Caucasian male). The skin donor is anonymous and informed consent was obtained by the supplier. NHDFs were grown at 37°C in an atmosphere of 5% CO2 in the *ad hoc* medium supplied by Clonetics (FGM-2 bulletKit system). Cells were seeded as recommended by the supplier and always split at 70% confluence. Normal human epidermal keratinocytes (NHEK) were purchased from Clonetics (CC-2501). We used cells from one donor referred to as K1MC. Cells were obtained anonymously and informed consent was obtained by the supplier. Cells were grown at 37°C in an atmosphere of 5% CO2 in a KGM-2 BulletKit medium consisting of modified MCBD153 with 0.15 mmol/L calcium, supplemented with bovine pituitary extract, epidermal growth factor, insulin, hydrocortisone, transferrin, and epinephrin (Clonetics). Cells were seeded at 3500 cells/cm^2 and always split at 70% confluence.

Antibodies & reagents

Sorafenib was purchased from Selleck chemicals and kept as 10 mM stock in DMSO at -20°C. Trametinib and rapamycin were purchased from Selleck chemicals. Tunicamycin, 4µ8C, GSK2606414 and puromycin were purchased from Calbiochem. All other compounds were purchased from Sigma. The following antibodies were used in this study.

Name	Species	provider	Reference
Puromycin	Mouse	Merk millipore	MABE 343
p-ERK (T202/Y204)	Rabbit	Cell signaling	9101
ERK	Rabbit	Cell signaling	4695
pAkt/PKB	Rabbit	Cell signaling	4060
Akt/PKB	Rabbit	Cell signaling	9272
pp70 S6K	Rabbit	Cell signaling	9234
p70 S6K	Rabbit	Cell signaling	2708
p-4EBP1 (T37/T46)	Rabbit	Cell signaling	2855
4EBP1	Rabbit	Cell signaling	9644
p-P38 (T180/Y182)	Rabbit	Cell signaling	9211
P38	Rabbit	Cell signaling	9212
p-RPS6 (S235/236)	Rabbit	Cell signaling	2211
RPS6	Rabbit	Cell signaling	2217
p-eIF2α (S51)	Rabbit	Cell signaling	3398
eIF2α	Rabbit	Santa Cruz	sc11386
sXBP-1	Rabbit	Cell signaling	12782
BIP/GRP78	Rabbit	Cell signaling	3177
Calreticulin	Rabbit	Abcam	ab2907
ERp57	Rabbit	Abcam	ab10287
PDI	Rabbit	Cell signaling	3501
RPL15	Rabbit	Genetex	GTX101830
RPL29	Rabbit	Genetex	GTX101833
RPSA	Rabbit	Genetex	GTX100831
eIF4A	Rabbit	Cell signaling	2013
eIF4E	Rabbit	Cell signaling	2067
eIF4G	Rabbit	Cell signaling	2469
eIF4H	Rabbit	Cell signaling	3469
β-Actin	Mouse	Sigma	A544R
Anti-mouse IgG-HRP linked	Sheep	GE Healthcare	NA931V
Anti-rabbit IgG-HRP linked	Donkey	GE Healthcare	NA934V

Metabolite extraction for NMR analysis

HCC cells (1.2×10^7 per condition) were rinsed with PBS and extracted in 6 ml of cold methanol. A dual-phase extraction: $MeOH/H_2O/CHCl_3$ (6/6/5.4) was performed and the aqueous phase was kept for analysis after pH adjustment to 7.4. Dried samples were dissolved in deuterated phosphate buffer (pH 7.4, 0.1 M) containing trimethyl-silyl-propionic acid sodium salt (TMSP 0.1 mM) as a standard calibration and NaN_3 (0.01 mM) (Martineau et al. 2011). This solution was homogenized and centrifuged at 20000 G at 4°C for 10 min, and the supernatant (600 µL) was transferred to a 5 mm NMR tube.

NMR spectra measurement

All NMR spectra were recorded on a Bruker Avance III 600 spectrometer operating at 600.13 MHz for ^1H, using a multinuclear broadband TXI 5 mm probe. The TOPSPIN 3.5 software (Bruker) was used for acquisition. The 1D spectra were collected using a solvent suppression pulse sequence based on a 1D Nuclear Overhauser Effect Spectroscopy (NOESY) pulse sequence to saturate the residual water signal [1]. For each spectrum, 32 free induction decay values (FIDs) were collected into 128 K data points using a spectral width of 8403 Hz with an acquisition time of 7.79 s. Two-dimensional J-resolved NMR spectra [2] were acquired with a 5.0 s relaxation delay using 4 transients per 64 increments that were collected into 64k data points, using spectral widths of 8417.5 Hz along the direct dimension (i.e., chemical shift axis) and 50 Hz along the indirect dimension (i.e., spin–spin coupling axis) and using excitation sculpting for the water suppression.

For all spectra, the FIDs were multiplied by an exponential weighting function corresponding to a line broadening of 0.3Hz and zero-filled before Fourier transformation. The 1D NMR spectra were exported to ASCII into "one point as bucket" using TopSpin 3.5 software. Data analysis was performed with the Matlab software (Matlab R2014b, The Mathworks Inc, Natick, MA, USA). The baseline was corrected with the Matlab package airPLS 2.0 [3]. Spectra were aligned with the "icoshift" program (v 1.2) [4,5]. The spectral assignments were based on matching the 1D and 2D data to standard reference spectra.

REFERENCES

1. Giraudeau P, Silvestre V, Akoka S. Optimizing water suppression for quantitative NMR-based metabolomics: a tutorial review. Metabolomics. 2015; 11:1041–1055. https://doi.org/10.1007/s11306-015-0794-7.
2. Ludwig C, Viant MR. Two-dimensional J-resolved NMR spectroscopy: review of a key methodology in the metabolomics toolbox. Phytochem Anal PCA. 2010; 21:22–32. https://doi.org/10.1002/pca.1186.
3. Zhang ZM, Chen S, Liang YZ. Baseline correction using adaptive iteratively reweighted penalized least squares. The Analyst. 2010; 135:1138–1146. https://doi.org/10.1039/b922045c.
4. Savorani F, Tomasi G, Engelsen SB. icoshift: A versatile tool for the rapid alignment of 1D NMR spectra. J Magn Reson. 2010; 202.
5. Tomasi G, Savorani F, Engelsen SB. icoshift: An effective tool for the alignment of chromatographic data. J Chromatogr A. 2011; 1218:7832–7840. https://doi.org/10.1016/j.chroma.2011.08.086.
